# Assessing the carotenoid profiles and allelic diversity of yellow maize inbred lines adapted to mid-altitude subhumid maize agroecology in Ethiopia

**DOI:** 10.3389/fpls.2024.1406550

**Published:** 2024-07-23

**Authors:** Belay Garoma, Girum Azimach, Kassahun Bante, Abebe Menkir

**Affiliations:** ^1^ Bako National Maize Research Center, Bako, Ethiopia; ^2^ EthioAgri-CEFT, Addis Ababa, Ethiopia; ^3^ College of Agriculture and Veterinary Medicine, Jima University, Jima, Ethiopia; ^4^ Maize Improvement Unit, International Institute of Tropical Agriculture, Ibadan, Nigeria

**Keywords:** biofortification, carotenoids profile, provitamin A, favorable allele, marker-assisted selection (MAS), yellow/orange maize inbred lines

## Abstract

Biofortification of provitamin A in maize is an attractive and sustainable remedy to the problem of vitamin A deficiency in developing countries. The utilization of molecular markers represents a promising avenue to facilitate the development of provitamin A (PVA)-enriched maize varieties. We screened 752 diverse tropical yellow/orange maize lines using kompetitive allele-specific PCR (KASP) makers to validate the use of KASP markers in PVA maize breeding. To this end, a total of 161 yellow/orange inbred lines, selected from among the 752 lines, were evaluated for their endosperm PVA and other carotenoid compounds levels in two separate trials composed of 63 and 98 inbred lines in 2020 and 2021, respectively. Significant differences (*p* < 0.001) were observed among the yellow maize inbred lines studied for all carotenoid profiles. An inbred line TZMI1017, introduced by the International Institute of Tropical Agriculture (IITA) showed the highest level of PVA (12.99 µg/g) and β-carotene (12.08 µg/g). The molecular screening showed 43 yellow maize inbred lines carrying at least three of the favorable alleles of the KASP markers. TZMI1017 inbred line also carried the favorable alleles of almost all markers. In addition, nine locally developed inbred lines had medium to high PVA concentrations varying from 5.11 µg/g to 10.76 µg/g and harbored the favorable alleles of all the KASP PVA markers. Association analysis between molecular markers and PVA content variation in the yellow/orange maize inbred lines did not reveal a significant, predictable correlation. Further investigation is warranted to elucidate the underlying genetic architecture of the PVA content in this germplasm. However, we recommend strategic utilization of the maize-inbred lines with higher PVA content to enhance the PVA profile of the breeding program’s germplasm.

## 1 Introduction

Maize is the most widely cultivated staple crop in Sub-Saharan Africa and provides over 20% of total calories in human diets (Badu-Apraku et al., 2020). In Ethiopia, maize is the second most widely cultivated staple crop after tef (*Eragrostis tef*), but it ranks first in terms of productivity and total production, with more than nine million households depending on it for their food and livelihoods (Abate et al., 2015). However, heavy dependence on the consumption of staple food crops like maize can contribute to micronutrient malnutrition such as vitamin A deficiency (VAD) (Menkir et al., 2018). VAD is prevalent in developing countries and is the main cause of preventable blindness and growth retardation in children and increased risk of maternal mortality in pregnant women (Olivia Pecukonis, 2017).

Biofortification of staple crops with essential micronutrients stands out as a cost-efficient and sustainable intervention against VAD (Gupta et al., 2015). Biofortification is the process of increasing the content of vitamins and minerals in the edible parts of staple crops through plant breeding techniques (Garg et al., 2018).

Maize biofortification efforts in Sub-Saharan Africa tackle the challenge of low provitamin A (PVA) content in local inbred lines (Parasanna et al., 2020). Breeders employed backcrossing, using high β-carotene temperate maize with elite white tropical inbred lines (Pixley et al., 2013); the inbred lines showed variation in carotenoid profiles (Babu et al., 2013; Sayadi Maazou et al., 2021). Conventionally, breeders relied on screening large numbers of genotypes for carotenoid profiles using high-performance liquid chromatography (HPLC) or ultra-performance liquid chromatography (UPLC) (Gupta et al., 2019). However, these techniques are expensive for routine PVA breeding programs. This is where molecular screening comes in—it allows researchers to detect alleles associated with high PVA content (Babu and Prasanna, 2014), offering a more cost-effective approach. Genetic markers, particularly for the *crtRB1* gene in the carotenoid biosynthesis pathway (Muthusamy et al., 2015), facilitated breeding for high-PVA maize. Particularly, favorable alleles at crtRB1-3’TE showed promise for increasing PVA content (Azmach et al., 2013; Babu et al., 2013). These findings suggest PVA-enriched inbred lines and associated markers are valuable tools. Recently developed kompetitive allele-specific PCR (KASP) markers targeting the gene *crtRB1* were reported to offer a reliable, high-throughput screening method for PVA content (Gowda et al., 2017). However, the genetic background in which these favorable alleles developed, population size, and marker–trait relationships may influence the effectiveness of such marker-assisted selection for main routine breeding programs (Babu et al., 2013).

In Ethiopia, yellow maize improvement was started in 2004 to meet the demand of the poultry industry, and subsequently, breeding for provitamin A enrichment for human nutrition began in 2008 through the introduction and evaluation of maize hybrids and inbred lines (Girum et al., 2012). Currently, the public maize breeding program in Ethiopia has developed a large number of yellow/orange maize inbred lines with diverse genetic backgrounds. In addition, the program has been introducing PVA inbred lines from IITA and CIMMYT and extensively evaluating them for various agronomic traits in multilocation field trials. Most of the locally developed yellow/orange inbred lines were derived from bi-parental crosses and backcrosses using a few temperate PVA trait donor inbred lines and locally adapted elite white inbred lines. These inbred lines have not been assayed for their provitamin A and other carotenoid contents in relation to their haplotype for the alleles of the *crtRB1* gene. Therefore, the objectives of our study were to (i) assess the PVA and other carotenoid profiles of the adapted yellow/orange maize inbred lines for use as parents to develop hybrids with high PVA content and source populations of new inbred lines; (ii) evaluate the inbred lines using a set of KASP markers associated with allelic variants of a major *crtRB1* gene; and (iii) select inbred lines carrying favorable alleles of high provitamin A content for subsequent use as parents to accelerate new PVA inbred line development using molecular markers.

## 2 Materials and methods

### 2.1 Plant materials

A total of 161 yellow/orange maize inbred lines along with checks (a locally developed white inbred line, BKL004, and a HarvestPluss inbred line, CLHP00003) were evaluated in two separate trials during the 2020 and 2021 main cropping seasons. The inbred lines had diverse genetic backgrounds, which include introductions from CIMMYT, IITA, and locally developed yellow/orange inbred lines. The local yellow/orange inbred lines at the F_5_ inbreeding stage were developed from crosses of adapted elite white maize inbred lines with introduced provitamin A donor lines through backcrossing and pedigree breeding methods.

### 2.2 Experimental site

The trials for carotenoid analysis and genotyping were planted at Bako Agricultural Research Center, Ethiopia. Bako represents the mid-altitude subhumid maize agroecology of Ethiopia. Its elevation is about 1,650 m above sea level and lies between 9°06′ north latitude and 37°09′ east longitude. Bako receives 1,200–1,500 mm of rainfall annually.

### 2.3 Analyses of carotenoids

Each of the two trials was composed of two sets of yellow/orange maize inbred lines with two checks; each trial was arranged in an alpha lattice design and blocks nested under two replications. Each inbred line was planted in one row with a row length of 5 m, a spacing of 75 cm, and a 25-cm distance between plants within a row. Trial I consisted of 63 inbred lines and was evaluated in the 2020 main cropping season, whereas trial II consisted of 98 inbred lines and was evaluated in the 2021 main cropping season.

Agronomic practices, including fertilizer application (NPS 150 kg/ha and UREA 250 kg/ha) and weed management, were carried out uniformly as per recommendation for the testing site.

Two plants were self-pollinated in each line to produce pure seeds for carotenoid analysis. Each ear was harvested separately, air-dried at ambient temperature, shelled, and stored in a cold room at 10°C for carotenoid analysis.

A hundred seed samples were drawn randomly from each plot and sent to CIMMYT, Mexico for carotenoid analysis. The carotenoid extraction and measurement were carried out as per the procedure CIMMYT’s laboratory manual (Muzhingi et al., 2017; Azmach et al., 2018).

Briefly, 600 mg of fine powder from each sample was transferred into a 15-mL glass tube container, to which 6 mL of ethanol with 0.1% of butylated hydroxyl toluene was added. Next, the tubes were shacked and vortexed for 15 s and incubated at 85°C for 5 min in a water bath. The tubes, which contain samples taken out from incubation and 500 μL of 80% potassium hydroxide added for saponification, were then shacked and vortexed.

Each tube sample was mixed with 3 mL of cold deionized water, 200 µL of internal standard β-Apo-80-carotenal, and 3 mL of hexane. The mixture was vortexed for 15 s, followed by a 3-min centrifugation at 3000 rpm. The supernatant (upper solvent phase) was pipetted into a 15-mL new test tube, placed on ice, and tightly covered. Again, 3 mL of hexane was added to each tube of sample and centrifuged. The supernatant (upper phase) was transferred to a new tube placed on ice and tightly covered.

Each extracted sample was dried under nitrogen gas using a Turbovap LV concentrator, reconstituted in 1 mL of 50:50 methanol:dichloroethane, and vortexed for 10 s. Using a mobile phase gradient from methanol:tert-butyl methyl ether (80:20 v/v) and a C30 Column (4.6 mm × 250 mm; 3 µm) to separate carotenoids, 50 µL of each sample was injected into the Acquity UPLC system. The solvent flow rate was 1 mL/min. The water acuity photodiode array detector is used to measure carotenoid absorbance at 450 nm wavelength (Muzhingi et al., 2017) and is connected to the software to view carotenoid profiles. Provitamin A was calculated as the sum of β-carotene and half of each of β-cryptoxanthin and α-carotene. Total carotenoid was calculated as the sum of PVA, lutein, and zeaxanthin. The concentration measurements were expressed in micrograms per gram of dry weight (DW).

### 2.4 Genotyping

The 752 inbred lines (**Supplementary Table S5**), which also contain the 161 inbred lines analyzed for their carotenoid content, were genotyped using seven KASP PCR markers with key allelic variants of the *crtRB1* gene. Each inbred line was planted on a single row of 5 m long at Bako Maize Research Center in the 2021 off-season. Young leaf samples were collected from five typical plants in each plot using labeled 96-well plates following the leaf sample collection procedure of the genotyping service provider. The leaf samples were dried in an oven at 50°C for 48 h, sealed, placed in a bucket containing silica gels, and stored in a cold room till samples were shipped to Sweden. DNA extraction protocol followed the CTAB method (Obi et al., 2020), and KASP PCR genotyping was carried out by Intertek AgriTech Laboratory, Sweden (https://www.intertek.com/agriculture/agritech/). The isolated DNA samples were genotyped using seven KASP markers presented in **Table 1**. PCR amplification and thermal cycling were performed according to the standard protocol for the KASP genotyping chemistry manual (https://www.biosearchtech.com/, accessed in 2020).

**Table 1:** KASP PCR markers were used to genotype 752 yellow/orange maize inbred lines.

SNP ID	Intertek ID	Owner	Trait category	Favorable allele	Unfavorable allele
S10–134583972	snpZM0013	CIMMYT	PVA	GG	CC
S10–134655704	snpZM0014	CIMMYT	PVA	CC	TT
SYN11355	snpZM0015	CIMMYT	PVA	AA	GG
PZE-110083653	snpZM0016	CIMMYT	PVA	GG	AA
S10–136072513	snpZM0017	CIMMYT	PVA	TT	GG
S10–136840485	snpZM0018	CIMMYT	PVA	CC	TT
S10–137904716	snpZM0019	CIMMYT	PVA	CC	TT

### 2.5 Statistical analysis

The carotenoid data were analyzed using PROC GLM in SAS software version 9.3 (SAS institute, 2011), in which inbred lines were treated as fixed effects while blocks and replications were treated as random effects. A mean separation was performed to compare treatment means using an LSD test at a 5% level of significance. Similarly, the repeatability (*R* ^2^) values for PVA and other carotenoids were estimated using the PROC GLM model using SAS software (version 9.3). The distribution of carotenoid traits was analyzed using *R* software version 3.1.4. Genotyping data of inbred lines were sorted and aligned in an Excel sheet to identify the presence of favorable alleles associated with provitamin A and other carotenoid profiles. The general linear model (GLM) was used to analyze the association of favorable alleles with carotenoid traits in the TASSEL 5.0 version (Bradbury et al., 2007).

## 3 Results

### 3.1 Carotenoid profile

**Figure 1:**
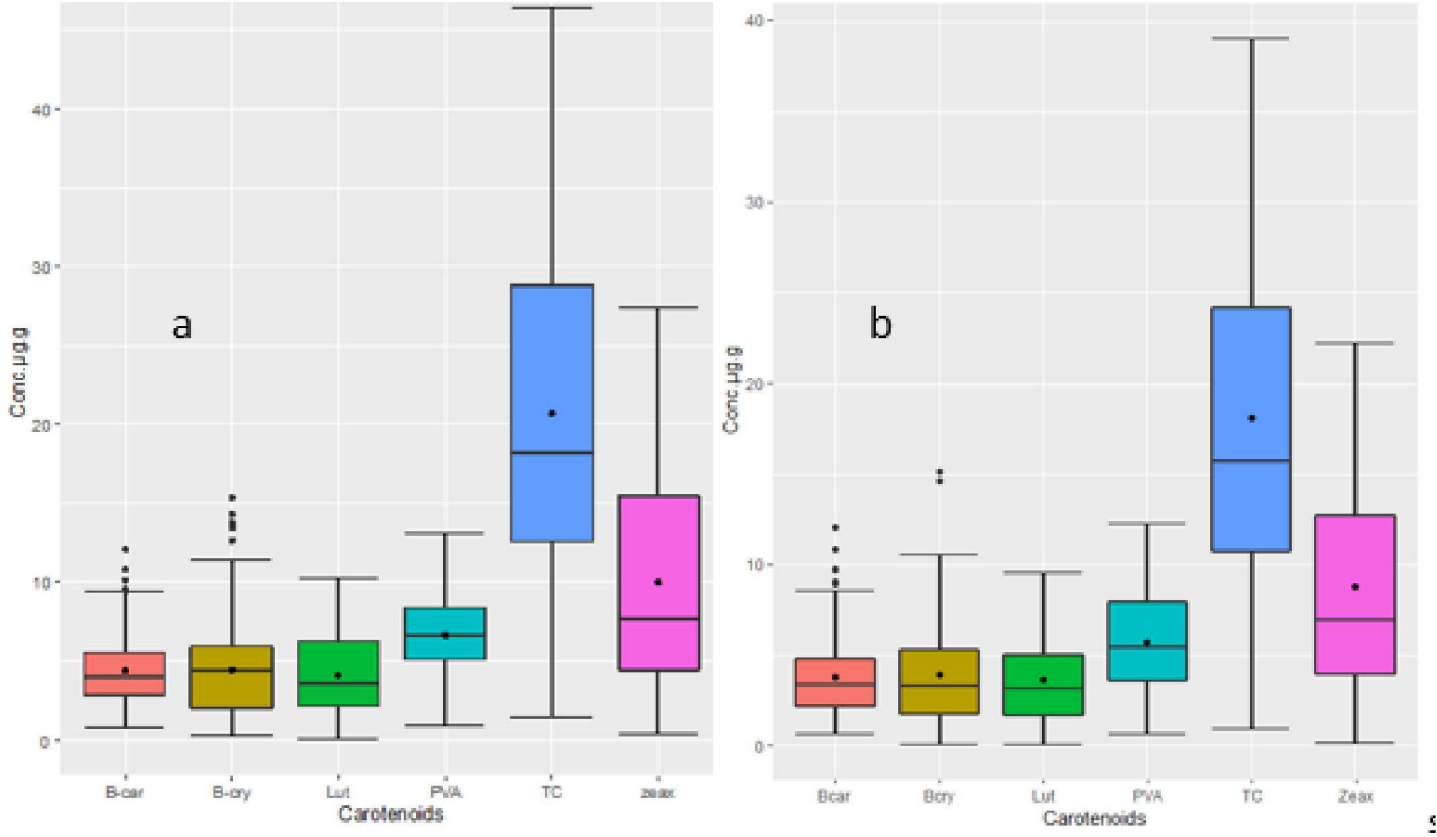
**(A)** Distribution of carotenoid concentrations for 98 inbred lines (Trial II). **(B)** Distribution of carotenoid concentrations for 63 inbred lines (Trial I). The upper and lower whisker (outside of the box plot) endpoints represent maximum and minimum concentrations, respectively. In the box plot, the lower and upper edges represent the first and third quartiles, the line inside the box represents the median, and the black point represents the mean. Carotenoids are abbreviated as follows: β-car, β-carotene; β-cry, cryptoxanthine; Lut, lutein; PVA, provitamin A; TC, total carotene; zeax, zeaxantine.

The analysis of variance showed highly significant differences (*p* < 0.001) among the yellow maize inbred lines in both trials I and II for PVA, β-carotene, β-cryptoxanthin, zeaxanthin, lutein, and total carotenoid concentrations (**Tables 2**, **3**). The distributions of PVA and other carotenoids (µg/g) for the two trials (trial I, 63 inbred lines, and trial II, 98 inbred lines) are presented in boxplots (**Figures 1A, B**, respectively). The two trials displayed a similar distribution of carotenoids among the inbred lines. A few out-layers were observed for β-carotene and β-cryptoxanthin in both trials.

**Table 2:** Mean squares for carotenoid content of 63 maize inbred lines evaluated in 2020.

Source of variation	*df*	Mean square of carotenoids					
		Bcar	β-Cry	PVA	Lutein	Zea	TC
Inbred lines	62	9.62^***^	16.98^***^	14.15^***^	10.33^***^	69.16^***^	164.85^***^
Rep	1	0.04	0.02	0.04	0.14	0.04	0.26
Block (rep)	4	0.12	0.11	0.10	0.12	0.40	2.10
Error	58	0.02	0.12	0.14	0.16	0.58	1.53

*** means highly significant.

**Table 3:** Mean squares for carotenoid content of 98 maize inbred lines evaluated in 2021.

Source of variation	*df*	Mean square of carotenoids					
		Bcar	β-Cry	PVA	Lutein	Zea	TC
Inbred lines	97	8.97^***^	14.38^***^	11.86^***^	10.27^***^	85.20^***^	187.36^***^
Rep	1	0.19	2.10	1.37	0.01	0.04	1.75
Block (rep)	12	0.59	0.67	0.51	0.38	3.78	5.65
Error	85	0.21	0.74	0.45	0.57	3.22	7.22

df, degree of freedom. *** means highly significant.

Repeatability (r) for all carotenoids ranged from 95% to 99% for trials I and II evaluated in 2020 and 2021, respectively.

The PVA content varied from 0.7 µg/g to 12.99 µg/g with a mean value of 6.64 µg/g; the β-carotene value varied from 0.65 to 12.07 with a mean of 4.41 µg/g and that of β-cryptoxanthin varied from 0.10 to 14.85 with a mean of 4.45 µg/g. Zeaxanthin showed the highest level ranging from 8.76 µg/g to 24.75 μg/g whereas lutein had the lowest level, ranging from 0.05 µg/g to 8.91 µg/g (**Supplementary Tables S1**, **S2**).

The inbred line TZMI1017, introduced from IITA, had the highest level of provitamin A (12.99 µg/g), followed by CML 297 (11.33 µg/g) and a locally developed inbred line (IL00’E-9–1-1–1-1–1/(KUI carotenoid syn-FS17–3-2-B-B-B/(KU1409/DE3/KU1409) S2–18-2-B)-B-1(MAS: L4H1)-5-B-B-B)-B-11–1-2-B (11.08 µg/g) (**Table 4**) and also supplementary (**Supplementary Table S6**) for all carotenoids. On the other hand, the two checks (the white seeded inbred line (BKL004) and HarvestPlus inbred line (CLHP00003) had the lowest provitamin A content, 0.7 µg/g and 1.08 µg/g, respectively. Inbred lines with mean PVA contents of 6.64 µg/g to 12.99 µg/g can be used as donor parents in PVA maize breeding.

**Table 4:** Selected inbred lines among 98 inbred lines based on their mean value of β-carotene and provitamin A content (µg/g).

Entry	Pedigree	β-Carotene	PVA
85	TZMI1017	12.08	12.99
3	IL00’E-9–1-1–1-1–1/(KUI carotenoid syn-FS17–3-2-B-B-B/(KU1409/DE3/KU1409) S2–18-2-B)-B-1(MAS: L4H1)-5-B-B-B)-B-11–1-2-B	10.13	11.08
7	(IL00’E-9–1-1–1-1–1/(KUI carotenoid syn-FS17–3-2-B-B-B/(KU1409/DE3/KU1409)S2–18-2-B)-B-1(MAS:L4H1)-5-B-B-B)-B-13–1-4-B-1	9.74	10.27
57	CML144/(KUI carotenoid syn-FS11–1-1-B-B-B/(KU1409/DE3/KU1409)S2–18-2-B)-B-3(MAS:L4H1)-1-B-B-B)-B-5–2-1	8.94	9.44
88	CLHP00306	8.13	10.60
54	(CML144/(KUI carotenoid syn-FS3–3-2-B-B-B(KU1409/DE3/KU1409)S2–18-2-B)-B-4(MAS:L4H1)-2-B-B-B)-B-9–1-1–1	8.01	9.18
49	(CML144/(KUI carotenoid syn-FS3–3-2-B-B-B(KU1409/DE3/KU1409)S2–18-2-B)-B-4(MAS:L4H1)-2-B-B-B)-B-3–2-4–1-1	7.91	10.76
86	TZMI1018	7.26	9.32
30	(DE78-Z-126–3-2–2-1–1(g)/(KUI carotenoid syn-FS11–1-1-B-B-B/(KU1409/DE3/KU1409) S2–18-2-B)-B-3(MAS: L4H1)-1-B-B-B)-B-1–1-1	6.76	9.17
4	(IL00’E-9–1-1–1-1–1/(KUI carotenoid syn-FS17–3-2-B-B-B/(KU1409/DE3/KU1409) S2–18-2-B)-B-1(MAS: L4H1)-5-B-B-B)-B-11–2-2	7.52	8.12
96	(CML486/(CML297-B<d7>KUICarotenoidsyn-FS17–3-2-B/KUI3<d7>B77))-B-11–1-B-B-B-B-B-B-B-#-B-B	6.10	10.32
94	CML 297	4.48	11.33
75	(Gibe1–91-1–1-1–1/(KUI carotenoid syn-FS17–3-2-B-B-B/(KU1409/DE3/KU1409)S2–18-2-B)-B-1(MAS:L4H1)-5-B-B-B)-B-2–2-1-B	5.76	8.88
90	CLHP00003, introduced from Harvest Plus (check)	0.90	1.10
84	BKL004, locally developed white maize inbred line check	0.92	1.09
	Mean	4.41	6.64
	CV	10.57	10.15
	LSD.05	0.93	1.34

### 3.2 Molecular screening

Out of the 752 diverse yellow maize inbred lines screened with the seven KASP markers, about 113 inbred lines were found to be carrying one favorable allele by one of the KASP markers, whereas 16 inbred lines carried two favorable alleles at least by two KASP PCR markers. Moreover, 43 inbred lines carried three or more favorable alleles of the *crtRB1* gene (**Supplementary Table S3**). Among 43 lines, 14 locally developed inbred lines carried favorable alleles of all markers of the *crtRB1* gene, suggesting that introgression of favorable alleles into adapted elite lines was successful. Inbred lines TZMI1017 and CML297, introduced and adapted to local conditions carried five and six favorable alleles of KASP PVA markers, respectively.

Inbred lines carrying the favorable alleles of the KASP markers and having high PVA content were identified. Inbred line TZMI1017 showed the highest levels of PVA (12.99 µg/g) and β-carotene (12.08 µg/g) and carried the favorable alleles of almost all the markers evaluated (**Table 5**). Inbred line CML297 had high PVA (11.32 µg/g) with high β-cryptoxanthine concentration and carried the favorable alleles by five KASP markers used. In addition, nine locally developed inbred lines had medium to high PVA concentrations (5.11 µg/g to 10.76 µg/g) and carried favorable alleles of the seven KASP markers (**Table 5**). However, the general linear model association analysis result showed that the KASP markers were not significantly associated with variation in PVA carotenoids, β-carotene, and β-cryptoxan (**Supplementary Table 4**).

**Table 5:** Selected inbred lines carrying favorable alleles of the *crtRB1* gene combined with high PVA content (µg g^−1^).

Sample ID coded	KASP SNP PVA markers							Carotenoids		
	snpZM00013	snpZM00014	snpZM00015	snpZM00016	snpZM00017	snpZM00018	snpZM00019	β-Car	β-Cry	PVA
ETBKCART110	C:C	T:T	A:A	G:G	T:T	C:C	C:C	4.01	4.75	6.39
ETBKCART113	G:G	C:C	A:A	G:G	T:T	C:C	C:C	1.64	1.55	2.42
ETBKCART137	G:G	C:C	A:A	G:G	N/A	C:C	C:C	3.37	4.40	5.55
ETBKCART143	G:G	C:C	A:A	G:G	T:T	C:C	T:T	3.04	5.90	5.97
ETBKCART153	G:G	C:C	A:A	G:G	T:T	C:C	C:C	4.01	7.25	7.63
ETBKCART154	G:G	C:C	A:A	N/A	T:T	N/A	C:C	3.18	4.10	5.23
ETBKCART157	G:G	C:C	A:A	G:G	T:T	C:C	C:C	7.91	5.70	10.76
ETBKCART168	G:G	C:C	A:A	G:G	T:T	C:C	T:T	2.32	5.60	5.11
ETBKCART169	G:G	C:C	A:A	N/A	T:T	C:C	T:T	3.13	5.35	5.78
ETBKCART177	G:G	C:C	A:A	A:A	T:T	N/A	C:C	3.93	7.90	7.88
ETBKCART193	G:G	C:C	A:A	G:G	T:T	C:C	C:T	12.08	1.85	12.99
ETBKCART202	G:G	C:C	A:A	N/A	T:T	C:C	T:T	4.48	13.70	11.33

green, favorable allele; blue, heterozygous; red, unfavorable allele; and yellow, not available/missed sample.

## 4 Discussion

Our result demonstrated high genetic variation among yellow maize inbred lines for all carotenoids, which was consistent with results in other studies (Muthusamy et al., 2015; Garg et al., 2018). Inbred lines with high β-carotene and PVA content were identified (**Table 4**). For example, inbred lines introduced from IITA and locally developed that contain PVA > 8 µg/g can be used as donor parents in hybrid and open variety development (Sayadi Maazou et al., 2021). The higher repeatability estimates, ranging from 95% to 99% for all carotenoid concentrations in both trials, indicate that the measured carotenoid values were reliable and the variation was mainly due to the genetic factors.

Different studies reported that genotype by environment interaction showed a minor and significant effect on PVA expression in maize. For instance, Menkir et al. (2008) and Muthusamy et al. (2015) reported that location and inbred lines by location interaction had a small fraction effect on lutein, α-carotene, and PVA. Similarly, there is a minor effect of genotype × environment interaction on lutein and zeaxanthin (Goswami et al., 2019a; Duo et al., 2021), whereas there is a nonsignificant effect on the PVA and β-carotene carotenoids of maize, indicating that β-carotene and PVA carotenoids are strongly influenced by genotypes. In the current study, we did not conduct line × year interaction, but the success of PVA breeding relies on the availability of genetic variation in yellow maize inbred lines (Menkir et al., 2008) and allelic diversity for PVA and other carotenoid content (Duo et al., 2021). On the other hand, variety × environment interaction showed effects on PVA content (Mengesha et al., 2019), and inbred lines × years of interaction showed significant effects on carotenoids except on β-carotene and PVA content (Menkir et al., 2015). Such inconsistent reports highlight that further investigation of genotype × environment interaction and other stress effects is required on PVA and other carotenoid content using a wide array of enriched yellow maize genetic backgrounds.

Marker-assisted selection is a useful tool to shorten the breeding cycle through the introgression of favorable alleles into agronomically superior elite lines to improve the nutritional quality of maize. We tested kompetitive allele-specific PCR (KASP) markers of the functional variations in the *crtRB1* gene of the carotenoid biosynthesis pathway in maize endosperm to screen diverse maize inbred lines adapted to the mid-altitude subhumid agro-ecology of Ethiopia. Association analysis using the GLM model showed a nonsignificant association between the KASP markers and variations in PVA contents, β-carotene, and β-cryptoxanthin in the set of maize inbred lines studied. This is contrary to the results of Sayadi Maazou et al. (2021), who reported that KASP markers had a significant association with β-carotene and PVA accumulation and a negative, nonsignificant association with β-cryptoxanthin in maize. This could be due to the genetic backgrounds of the local inbred lines than those used in developing KASP markers, as that can affect marker–trait association (Babu et al., 2013). Likewise, the functional markers’ predictive accuracy seemed variable depending on the source of genotype and the combination of favorable alleles (Gedil and Menkir, 2019). Our results from similar studies (Esuma et al., 2022; Codjia et al., 2023) reported only one marker showed a significant association with PVA accumulation in cassava. Similarly, Vignesh et al. (2012) and Gebremeskel et al. (2018) reported that some lines carrying unfavorable alleles of the *crtRB1* gene expressed high levels of PVA content, which might be due to *ZmBCH1* and other genes. Such marker-to-trait association inconsistencies within a set of genotypes require further study, including phenotyping a larger sample of inbred lines and also checking for additional marker systems.

## 5 Conclusion

The genetic variation observed in PVA and other carotenoids among the inbred lines suggests their potential value for use as parents to enhance the PVA content through crossing, recycling, and strategic selection. The KASP markers examined in this study did not show a clear, predictable association with the observed PVA variation. However, it is crucial to continue investigating the underlying cause and test a more comprehensive marker system to streamline the regular PVA maize breeding program.

## Data availability statement

The original contributions presented in the study are included in the article/**Supplementary Material**. Further inquiries can be directed to the corresponding author.

## Author contributions

BG: Data curation, Formal analysis, Software, Writing – original draft, Methodology. GA: Conceptualization, Methodology, Visualization, Writing – review & editing, Project administration. KB: Supervision, Visualization, Validation, Writing – review & editing. AM: Funding acquisition, Project administration, Writing – review & editing.

## Supplementary material

Supplementary Table 1 Mean, minimum, and maximum distribution of carotenoids (µg/g) in2021.

Supplementary Table 2 Mean, minimum, and maximum distribution of carotenoids (µg/g) in 2020.

Supplementary Table 3 Genotypes of 43 provitamin A maize inbred lines carrying three or more favorable alleles of the seven crtRB1-KASP PCR markers.

Supplementary Table 4 Association of the presence of favorable alleles of KASP markers with mean of β-carotene, β-cryptoxanthine.

Supplementary Table 5 Provitamin A in inbred lines. A list of inbred lines used for genotyping.

Supplementary Table 6 Mean value of PVA and other carotenoid content (µg/g) among 98 yellow inbred lines.

## References

[B1] Abate T. Shiferaw B. Menkir A. Wegary D. Kebede Y. Tesfaye K.. (2015). Factors that transformed maize productivity in Ethiopia. Food Secur. 7, 965–981. doi: 10.1007/s12571-015-0488-z

[B2] Azmach G. Gedi M. Mendkir A. Spillane C. (2013). Marker-trait association analysis of functional gene markers for provitamin A levels across diverse tropical yellow maize inbred lines. BMC Plant Bio 13, 227–242. doi: 10.1186/1471-2229-13-227 24373137 PMC3890589

[B3] Azmach G. Menkir A. Spillane C. Gedil M. (2018). Genetic loci controlling carotenoid biosynthesis in diverse tropical maize lines. Genes Genomes Genet. 8, 1049–1065. doi: 10.1534/g3.117.300 PMC5844293 29378820

[B4] Babu R. Prasanna B. M. (2014). “Molecular breeding for quality protein maize (QPM),” in Genomics of plant genetic resources. Eds. Tuberosa R. Graner A. Frison E. (Springer Dordrecht Heidelberg, New York, London), 489–505.

[B5] Babu R. Rojas N. P. Gao S. Yan J. Pixley K. (20132013). Validation of the effects of molecular marker polymorphisms in LcyE and CrtRB1 on provitamin A concentrations for 26 tropical maize populations. Theor. Appl. Genet. 126, 389–399. doi: 10.1007/s00122-012-1987-3 23052023 PMC3555234

[B6] Badu-Apraku B. Fakorede B. Talabi A. O. Obeng-Bio E. Tchala N. Oyekale S. A. (2020). “Quantitative genetics, molecular techniques and agronomic performance of provitamin a maize in sub-Saharan Africa,” in Quantitative genetics, genomics and plant breeding (CABI, Wallingford UK), 276–324.

[B7] Bradbury P. J. Zhang Z. Kroon D. E. Casstevens T. M. Ramdoss Y. Buckler E. S. (2007). TASSEL: software for association mapping of complex traits in diverse samples. Bioinformatics 23, 2633–2635. doi: 10.1093/bioinformatics/btm308 17586829

[B8] Codjia E. D. Olasanmi B. Ugoji C. E. Rabbi L. Y. (2023). SNP-based marker-assisted selection for high provitamin A content in African cassava genetic background. South Afr. J. Sci. 119, 1–10. doi: 10.17159/sajs.2023/15115

[B9] Duo H. Hossain F. Muthusamy V. Zanjeer R. U. Goswami R. Chand G.. (2021). Development of sub-tropically adapted diverse provitamin-A rich maize inbreds through marker-assisted pedigree selection, their characterization and utilization in hybrid breeding. PLoS One 16, e0245497. doi: 10.1371/journal.pone.0245497 33539427 PMC7861415

[B10] Esuma W. Eyoo O. Gwandu F. Mukasa S. Alicai T. Ozimati A.. (2022). Validation of KASP markers associated with cassava mosaic disease resistance, storage root dry matter and provitamin A carotenoid contents in Ugandan cassava germplasm. Front. Plant Sci. 13, 1017275. doi: 10.3389/fpls.2022.1017275 36507387 PMC9727383

[B11] Garg M. Sharma N. Sharma S. Kapoor P. Kumar A. Chunduri V.. (2018). Biofortified crops generated by breeding, agronomy, and transgenic approaches are improving lives of millions of people around the world. Front. Nutr. 5, 12. doi: 10.3389/fnut.2018.00012 29492405 PMC5817065

[B12] Gebremeskel S. Garcia-Oliveira A. L. Menkir A. Adetimirin V. Gedil M. (2018). Effectiveness of predictive markers for marker assisted selection of pro-vitamin A carotenoids in medium-late maturing maize (Zea mays L.) inbred lines. J. Cereal Sci. 79, 27–34. doi: 10.1016/j.jcs.2017.09.001

[B13] Gedil M. Menkir A. (2019). An integrated molecular and conventional breeding scheme for enhancing genetic gain in maize in Africa. Front. Plant Sci. 10, 1430. doi: 10.3389/fpls.2019.01430 31781144 PMC6851238

[B14] Girum A. Mosisa W. Legesse W. Wende A. Berhanu T. (2012). Development of improved yellow maize germplasm in Ethiopia. Eds. Worku M. Twumasi-Afriyie S. Wolde L. Tadesse B. Demisie G. (Addis Ababa, Ethiopia: EIAR/CIMMYT), 58–65. Meeting the Challenges of Global Climate Change and Food Security through Innovative Maize Research. Proceedings of the Third National Maize Workshop of Ethiopia April 18–20, 2011.

[B15] Goswami R. Zunjare R. U. Khan S. Muthusamy V. Baveja A. Das A. K. (2019a). Genetic variability of kernel provitamin-A in sub-tropically adapted maize hybrids possessing rare allele of β-carotene hydroxylase. Cereal Res. Commun. 47, 205–215. doi: 10.1556/0806.47.2019.12

[B16] Gowda M. Worku M. Nair S. K. Palacios-Rojas N. Prasanna B. M. (2017). “Quality assurance/quality control (QA/QC) in maize breeding and seed production,” in Theory and Practice (CIMMYT, Nairobi, Kenya).

[B17] Gupta H. S. Hossain F. Muthusam V. (2015). Biofortification of maize: An Indian perspective. Indian J.Genet. 75, 1–22. doi: 10.5958/0975-6906.2015.00001.2

[B18] Gupta H. S. Hossain F. Muthusamy V. Zunjare R. U. (2019). “Marker-assisted breeding for enrichment of provitaminA in maize,” in Quality breeding in field crops (Springer, Cham), 139–157.

[B19] Mengesha W. Menkir A. Meseka S. Bossey B. Afolabi A. Burgueno J.. (2019). Factor analysis to investigate genotype and genotype× environment interaction effects on pro-vitamin A content and yield in maize synthetics. Euphytica 215, 1–15. doi: 10.1007/s10681-019-2505-3

[B20] Menkir A. Liu W. White W. S. Maziya-Dixon B. Rocheford T. (2008). Carotenoid diversity in tropical-adapted yellow maize inbred lines. Food Chem. 109, 521–529. doi: 10.1016/j.foodchem.2008.01.002

[B21] Menkir A. Natalia P. Oladeji A. Maria Cristina D. Thanda D. Bussie M.. (2018). VitaminA-Biofortified Maize: Exploiting Native Genetic Variation for Nutrient Enrichment. Science Brief: Biofortification No. 2 (HarvestPlus, and Crop Trust (Bonn, Germany: Science Brief: Biofortification).

[B22] Menkir A. Rocheford T. Maziya-Dixon B. Tanumihardjo S. (2015). Exploiting natural variation in exotic germplasm for increasing provitamin-A carotenoids in tropical maize. Euphytica 205, 203–217. doi: 10.1007/s10681-015-1426-z

[B23] Muthusamy V. Hossain F. Thirunavukkarasu N. Saha S. Gupta H. S. (2015). Allelic variations for *lycopene ϵ-cyclase*and *β-carotene hydroxylase* genes in maize inbreds and their utilization in β-carotene enrichment programme. Cogent Food Agric. 1, 1. doi: 10.1080/23311932.2015.1033141

[B24] Muzhingi T. Palacios N. Miranda A. Cabrera M. L. Yeum K. J. Tang G. (2017). Genetic variation of carotenoids, vitamin E and phenolic compounds in biofortified maize. J. Sci. Food Agric. 1–9. doi: 10.1002/jsfa.7798 27173638

[B25] Obi Q. N. Menkir A. Babalola D. F. Gedil M. (2020). Development of Efficient Genotyping Workflow for Accelerating Maize Improvement in Developing Countries. (Research square) 1–22. doi: 10.21203/rs.3.rs-129326/v1 PMC10313903 37398911

[B26] Olivia Pecukonis (2017). Global data on vitamin A status is critical for program decision but often outdated, reveals new study. Available online at: https://www.nutritionintl.org/news/.

[B27] Parasanna B. M. Palacios-Rojas N. Hossain F. Muthusamy V. Menkir A. Dhliwayo T.. (2020). Molecular breeding for nutritionally enriched maize: status and prospects. Front. Genet. 10. doi: 10.3389/fgene.2019.013924 PMC7046684 32153628

[B28] Pixley K. Palacios N. R. Babu R. Mutale R. Surles R. Simpungwe E. (2013). “Biofortification of maize with provitamin A carotenoids,” in Carotenoids in Human Health. Ed. Tanumihardo S. A. (inger Science and Business Media, New York), 271–292. doi: 10.1007/978-1-62703-203-2

[B29] SAS institute (2011). The SAS system for windows. Version 9.3 (Cary, NC, United States: SAS Inst).

[B30] Sayadi Maazou A. R. Gedil M. Adetimirin V. O. Meseka S. Mengesha W. Babalola D.. (2014). Comparative assessment of effectiveness of alternative genotyping assays for characterizing carotenoids accumulation in tropical maize inbred lines. Agronomy 11 (10), 2022.

[B31] Vignesh M. Nepolean T. Hossain F. Singh A. K. Gupta H. S. (2013). Sequence variation in 3′ UTR region of crtRB1 gene and its effect on β-carotene accumulation in maize kernel. J. Plant Biochem. Biotechnol. 22, 401–408. doi: 10.1007/s13562-012-0168-4

