# Supplementary material for: Assessing the carotenoid profiles and allelic diversity of yellow maize inbred lines adapted to mid-altitude subhumid maize agroecology in Ethiopia
Source: Front Plant Sci. 2024 Jul 23;15:1406550. doi: 10.3389/fpls.2024.1406550 (PMC11300288; doi:10.3389/fpls.2024.1406550)
Supplement: Supplementary Table 1 — Mean, minimum, and maximum distribution of carotenoids (µg/g) in2021. [file Table_1.docx]

Supplementary Table S1: Mean, minimum and maximum distribution of carotenoids (µg/g) in2021

| Traits | Minimum | Mean | Maximum |
| --- | --- | --- | --- |
| PVA | 1.08 | 6.64 | 12.99 |
| BC | 0.90 | 4.41 | 12.07 |
| BXC | 0.35 | 4.45 | 14.85 |
| Zn | 0.95 | 9.93 | 24.75 |
| Lt | 0.10 | 4.10 | 9.25 |
| TC | 1.66 | 20.69 | 42.19 |

Supplementary Table S2. Mean, minimum and maximum distribution of carotenoids (µg/g) in 2020

| Traits | Minimum | Mean | Maximum |
| --- | --- | --- | --- |
| PVA | 0.70 | 5.74 | 11.63 |
| BC | 0.65 | 3.78 | 11.46 |
| BXC | 0.10 | 3.94 | 14.86 |
| Zn | 0.18 | 8.76 | 22.10 |
| Lt | 0.05 | 3.61 | 8.91 |
| TC | 0.94 | 18.12 | 37.41 |
