# Supplementary material for: Assessing the carotenoid profiles and allelic diversity of yellow maize inbred lines adapted to mid-altitude subhumid maize agroecology in Ethiopia
Source: Front Plant Sci. 2024 Jul 23;15:1406550. doi: 10.3389/fpls.2024.1406550 (PMC11300288; doi:10.3389/fpls.2024.1406550)
Supplement: Supplementary Table 2 — Mean, minimum, and maximum distribution of carotenoids (µg/g) in 2020. [file Table_2.docx]

Supplementary Table S3. Genotypes of 43 Provitamin A maize inbred lines carrying three or more favorable alleles of the seven crtRB1-KASP PCR markers.

| Sample ID | snpZM00013 | snpZM00014 | snpZM00015 | snpZM00016 | snpZM00017 | snpZM00018 | snpZM00019 |
| --- | --- | --- | --- | --- | --- | --- | --- |
| ETBKCART12 | G: G | C:C | A: A | G: G | T: T | C:C | C:C |
| ETBKCART60 | C:C | T: T | A: A | G: G | T: T | C:C | C:C |
| ETBKCART61 | C:C | T: T | A: A | G:G | T: T | C:C | C:C |
| ETBKCART62 | C:C | T: T | A: A | N/A | T: T | C:C | N/A |
| ETBKCART63 | G: G | C:C | A: A | N/A | T: T | C:C | C:C |
| ETBKCART64 | G: G | C:C | A: A | G: G | T: T | C:C | C:C |
| ETBKCART98 | G: G | C:C | A: A | G: G | T: T | C:C | C:C |
| ETBKCART99 | G: G | C:C | A: A | G: G | T: T | C:C | C:C |
| ETBKCART101 | G: G | C:C | A: A | G:G | T: T | C:C | C:C |
| ETBKCART102 | G: G | C:C | A: A | N/A | T: T | C:C | C:C |
| ETBKCART110 | C:C | T: T | A: A | G: G | T: T | C:C | C:C |
| ETBKCART113 | G: G | C:C | A: A | G: G | T:T | C:C | C:C |
| ETBKCART137 | G: G | C:C | A: A | G: G | N/A | C:C | C:C |
| ETBKCART143 | G: G | C:C | A: A | G: G | T: T | C:C | T: T |
| ETBKCART153 | G: G | C:C | A: A | G:G | T: T | C:C | C:C |
| ETBKCART154 | G: G | C:C | A: A | N/A | T: T | N/A | C:C |
| ETBKCART157 | G: G | C:C | A: A | G: G | T: T | C:C | C:C |
| ETBKCART168 | G: G | C:C | A: A | G:G | T: T | C:C | T:T |
| ETBKCART169 | G: G | C:C | A: A | N/A | T: T | C:C | T:T |
| ETBKCART177 | G: G | C:C | A: A | A:A | T: T | N/A | C:C |
| ETBKCART192 | G:G | C:C | A:A | G:G | T:T | C:C | C:T |
| ETBKCART193 | G: G | C:C | A: A | N/A | T: T | C:C | T: T |
| ETBKCART202 | C:C | T: T | A: A | G: G | T: T | C:C | C:C |
| ETBKCART208 | G: G | C:C | A: A | G:G | T: T | C:C | T: T |
| ETBKCART233 | G: G | C:C | A: A | N/A | T: T | C:C | C:C |
| ETBKCART235 | G: G | C:C | A: A | G: G | T: T | C:C | C:C |
| ETBKCART248 | G: G | C:C | A: A | G: G | T: T | N/A | C:C |
| ETBKCART249 | G: G | C:C | A: A | G: G | T: T | N/A | C:C |
| ETBKCART318 | G: G | C:C | A: A | G: G | T: T | N/A | T: T |
| ETBKCART357 | G: G | C:C | A: A | G: G | T: T | C: T | C:C |
| ETBKCART562 | G: G | C:C | A: A | G:G | T: T | C:C | T: T |
| ETBKCART603 | C:C | T: T | G: G | N/A | T: T | C:C | C:C |
| ETBKCART632 | C:C | T: T | G: G | G: G | T: T | C:C | C:C |
| ETBKCART633 | C:C | T: T | A: A | G: G | T: T | C: T | C:C |
| ETBKCART639 | G: G | C:C | A: A | G:G | T: T | C:C | C:C |
| ETBKCART684 | G: G | C:C | A:A | N/A | T:T | C:C | C: T |
| ETBKCART696 | G: G | C:C | G: A | A: A | G: T | C:C | C: T |
| ETBKCART698 | G: G | C:C | A: A | N/A | T: T | C:C | C: T |
| ETBKCART699 | G: G | C:C | A: A | N/A | T: T | C:C | T: T |
| ETBKCART700 | G: G | C:C | A: A | G: G | T: T | C:C | C:C |
| ETBKCART702 | G: G | C:C | A: A | G: G | T: T | C:C | C:C |
| ETBKCART708 | G: G | C:C | A: A | G: G | T: T | C:C | C:C |
| ETBKCART709 | G: G | C:C | A: A | G: G | T:T | C:C | C:C |

Notice: green= favorable allele; blue = heterozygous, red = unfavorable allele and yellow color= Not available or missed sample
