# Supplementary material for: Assessing the carotenoid profiles and allelic diversity of yellow maize inbred lines adapted to mid-altitude subhumid maize agroecology in Ethiopia
Source: Front Plant Sci. 2024 Jul 23;15:1406550. doi: 10.3389/fpls.2024.1406550 (PMC11300288; doi:10.3389/fpls.2024.1406550)
Supplement: Supplementary Table 3 — Genotypes of 43 provitamin A maize inbred lines carrying three or more favorable alleles of the seven crtRB1-KASP PCR markers. [file Table_3.docx]

Supplementary Table S4. Association of the presence of favorable alleles of KASP markers with mean of β-carotene, β-cryptoxanthine and Provitamin A in inbred lines

| KASP PCR markers | β-carotene | | β-cryptoxanthine | | Provitamin A | |
| --- | --- | --- | --- | --- | --- | --- |
|  | P | R2 | P | R2 | P | R2 |
| snpZM0013 | 0.89 | 0.00191 | 0.07 | 0.03238 | 0.28 | 0.0119 |
| snpZM0014 | 0.98 | 0.00296 | 0.10 | 0.0273 | 0.37 | 0.0081 |
| snpZM0015 | 0.58 | 0.00307 | 0.38 | 0.00784 | 0.98 | 0.0017 |
| snpZM0016 | 0.87 | 0.00298 | 0.87 | 0.0034 | 0.95 | 0.0034 |
| snpZM0017 | 0.71 | 0.00142 | 0.29 | 0.01207 | 0.80 | 0.0064 |
| snpZM0018 | 0.86 | 0.003229 | 0.72 | 0.00138 | 0.97 | 0.0014 |
| snpZM0019 | 0.89 | 0.00173 | 0.87 | 0.00253 | 0.97 | 0.0079 |
